# The mediating role of postpartum depression between mother-infant contact barriers and maternal attachment: a cross-sectional study from Turkey

**DOI:** 10.1590/1806-9282.20241413

**Published:** 2025-03-31

**Authors:** Sümeyra Topal, Sinem Yalnizoğlu Çaka

**Affiliations:** 1 Kahramanmaraş İstiklal University, Faculty of Health Sciences, Department of Pediatric Nursing – Kahramanmaraş, Turkey.; 2 Kocaeli University, Faculty of Health Sciences, Department of Pediatric Nursing – Kocaeli, Turkey.

**Keywords:** Mother, Post natal depression, Attachment, Nursing care

## Abstract

**OBJECTIVE:**

The aim of this study was to investigate the role of postpartum depression in the relationship between mother-infant contact barriers and maternal attachment in mothers of premature infants.

**METHODS:**

This cross-sectional study was conducted on 113 mothers who applied to the pediatric outpatient clinic of a hospital between April and November 2022 and whose premature babies were discharged at least 1 month ago. The data were collected using the Edinburgh Postnatal Depression Scale, the Mother-Infant Contact Barriers Scale, and the Maternal Attachment Scale.

**RESULTS:**

The mothers’ mean Maternal Attachment Scale score was 82.25±10.57, the mean Mother-Infant Contact Barriers Scale score was 60.65±17.87, and the mean Edinburgh Postnatal Depression Scale score was 18.41±8.38. The mean Edinburgh Postnatal Depression Scale score of 78.8% (n=89) of the mothers was 14 and above. There was a statistically significant positive effect of the Mother-Infant Contact Barriers Scale on the Edinburgh Postnatal Depression Scale (B=0.95, p<0.001) and a statistically significant negative effect of the Edinburgh Postnatal Depression Scale on the Maternal Attachment Scale (B=-0.29, p<0.001). Hence, it was found that the Edinburgh Postnatal Depression Scale (B=-0.27, p<0.001) played a mediating role in the impact of the Mother-Infant Contact Barriers Scale on the Maternal Attachment Scale.

**CONCLUSION:**

Accordingly, it can be stated that maternal attachment decreases and postpartum depression increases as mother-infant contact barriers increase.

## INTRODUCTION

Mothers of preterm infants usually grieve the loss of a perceived "ideal pregnancy" while coping with a sick baby who needs additional medical care^1^. Mothers may be psychologically unprepared because preterm birth is usually unexpected^2^ and the mother-infant separation is prolonged^3^. If the infant, whom they have dreamed of being born healthy, is born preterm or is seriously ill, the mother may experience various negative emotions, such as shock, anxiety, grief, guilt or shame, feeling of helplessness, delay in grasping the reality of the situation, despair, anger, blaming the health personnel, constant crying, excessive silence, and inactivity^1,2,4,5^. This situation may adversely impact the mother's perception of and attachment with her infant.

Maternal sadness and postnatal depression (PND) are mental health problems in the postpartum period^6^. The prevalence of PND in all childbirths varies between 10 and 15%, depending on the screening tool used, timing, and cutoff scores, whereas this rate almost doubles (28 and 40%) in mothers of preterm infants^5,7^. PND can disrupt the mother-infant interaction, causing insecure attachment, developmental delay, and social interaction difficulties in affected children^8^. Since the mother is not ready for her infant to be born preterm, she may have difficulty caring for the preterm infant. Moreover, mothers feel alien to their infants^8^. It is known that families of preterm infants are less self-confident about their parenting competencies at home than families of term infants, and they prefer to follow care rather than actively taking part in the care of the infant^8,9^. Maternal attachment, a unique love relationship that starts in the first days of life and develops over time between the mother and the infant, can be impacted by the mother's psychological and social condition in the postpartum period, thus preventing secure mother-infant attachment^9^. It is known that the factors that prevent the mother and her infant from establishing contact have adverse impacts on the emotional, behavioral, and cognitive development of the infant and the attachment pattern^9,10^. This study was conducted to determine the relationship between PND, mother-infant contact barriers, and maternal attachment in mothers of preterm infants.

## METHODS

### Study design and participants

The current research is a descriptive and cross-sectional study. Data were collected from mothers who applied to the pediatric outpatient clinics of a hospital in Kahramanmaraş province between April and November 2022. Power analysis was conducted using the G*Power (3.1.9.2) program in order to determine the sample size. The sample size was determined as 86 mothers according to the effect size value of 0.34 and when Type 1 error probability (α) was accepted as 0.05 and Type 2 error probability (β) was accepted as 0.10 (at the 90% power level) for the determination of the relationship between the Maternal Attachment Scale (MAS) and the Mother-Infant Contact Barriers Scale (MICBS) obtained from the literature^10^. The study population consisted of all mothers of preterm infants who were discharged from the hospital between the data collection dates, while the sample comprised mothers (n=113) who voluntarily agreed to take part in the research at the date of data collection, who were 19 years of age and older, who did not have any pre-diagnosed psychiatric disorder, whose infants were discharged from the hospital, and who were at home for at least 1 month.

### Instruments

In this study, data were collected using a questionnaire prepared by the researchers, the Edinburgh Postnatal Depression Scale (EPDS), the MAS, and the MICBS. The questionnaire, developed by the researchers in accordance with the literature, contained 23 questions on the sociodemographic characteristics of mothers and their infants. The EPDS is a 4-point Likert self-evaluation scale, which was developed by Cox et al.^11^ and whose validity and reliability study was performed by Engindeniz et al.^12^. The cutoff point for the EPDS was computed as 13, and women with a total scale score above the cutoff point are considered the risk group. In the validity and reliability analysis of the scale, Cronbach's alpha coefficient was 0.79^12^, and it was found to be 0.96 for this study. The MAS, developed by Muller and Mercer^13^ to reveal the level of attachment between the mother and the infant and adapted to Turkish by Kavlak and Şirin^14^, has 26 statements that individuals can use to describe their feelings. A high score demonstrates high maternal attachment. In the validity and reliability analysis of the scale, the Cronbach's alpha coefficient was 0.72 for mothers with a 4-month-old infant, and it was found to be 0.95 for this study. The MICBS, developed by Akik et al. to measure possible contact barriers between the mother and the infant based on the mother's statement, is a 5-point Likert scale comprising 18 items^10^. The higher the total score from the scale is, the higher the contact barriers between the mother and the infant are. The Cronbach's alpha coefficient was 0.81 in the original version of the scale, and it was found to be 0.94 for this study.

### Ethics statement

Before the study commencement, ethics committee approval was acquired from the Ethics Committee, Faculty of Medicine, Kahramanmaraş Sütçü İmam University (approval number: 03, date: 22.03.2022), and necessary permissions were acquired from the hospital. The study adheres to the principles of the Helsinki Declaration. All subjects provided informed consent prior to data collection.

### Statistical analysis

**Figure 1:**
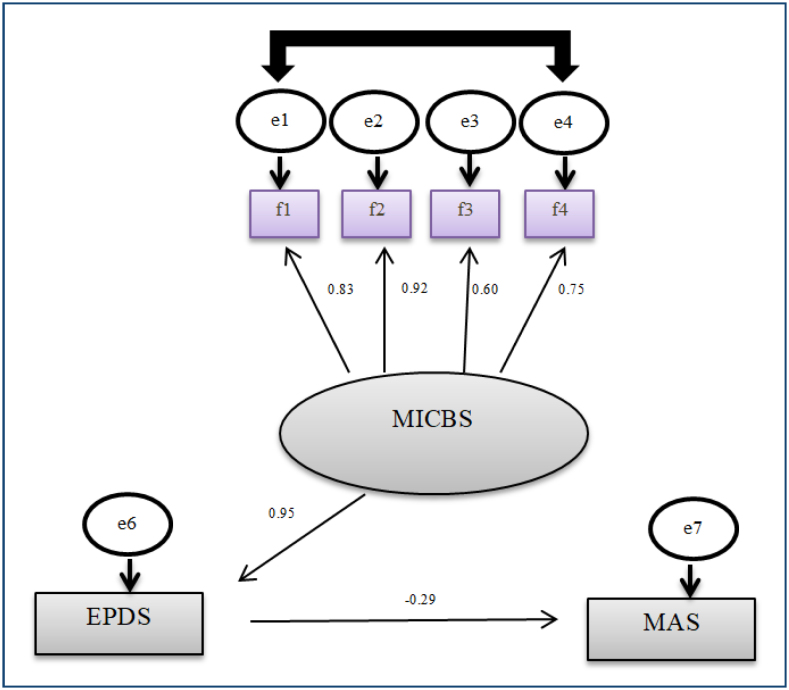
A conceptual pathway analysis model for the Edinburgh Postnatal Depression Scale, Mother-Infant Contact Barriers Scale, and Maternal Attachment Scale in mothers (n=113). f1: Postpartum physical contact barriers; f2: Mother-infant relationship and harmony difficulties; f3: Negative experiences regarding birth; f4: Positive emotions regarding postpartum first contact.

The study data were computed using the IBM SPSS Amos 23 program. Pearson's correlation analysis was employed in the analysis of the relationship between the scales, and path analysis was utilized in the analysis of the model. In the first stage, the path model in which the four-factor dimension of the MICBS is included as an indicator variable and the EPDS is included as a mediator variable is designed as shown in Figure 1. Since latent variables are not metric, to estimate parameter values, one of the paths drawn from latent variables to observed (indicator) variables must be assigned a value of 1 (factor loading equal to 1), or a value must be assigned to the variance of the latent variable (usually 1). In the second stage, the maximum likelihood method, which is frequently used in structural equation models and provides reliable results even in cases where the data is not normally distributed, was used while estimating the model, and it was aimed to estimate the parameters including the errors of the observed variables, the variances of the latent variables, and the regression coefficients related to the paths drawn from the latent variables to the observed variables. In the last stage, the fit indices for the created path model were examined. A value of p<0.05 was accepted for significance.

## RESULTS

Table 1 summarizes the descriptive characteristics of the 113 participating mothers in the study.

**Table 1:** Comparison of the differences between the scales and sociodemographic variables by the groups (n: 113).

Variables		n	%	MAS	MICBS	EPDS
				Mean±SD	Mean±SD	Mean±SD
Planned pregnancy	Yes	53	46.9	87.6±10.87	60.40±18.00	18.43±8.49
	No	60	53.1	83.17±9.93	60.87±17.90	18.38±8.35
t/p				**2.268/0.025** *	-0.139/0.890	0.032/0.975
Infant's sex	Female	49	43.4	85.95±10.11	63.47±17.53	19.10±8.14
	Male	64	56.6	84.70±10.96	58.48±17.96	17.87±8.58
t/p				0.624/0.534	1.447/0.142	0.770/0.443
Mode of delivery	Vaginal birth	32	28.3	86.94±10.12	64.00±14.51	19.66±6.31
	Cesarean section	81	71.7	84.58±10.73	59.32±18.95	17.91±9.06
t/p				1.069/0.288	1.410/0.163	1.160/0.250
Infant's birth week	24-29	62	54.9	83.34±9.82^b^	73.13±6.49^a^	24.26±3.11^a^
	30-32	32	28.3	83.88±9.43^b^	56.19±7.42^b^	16.38±2.98^b^
	33-37	19	16.8	93.79±11.06^a^	27.42±3.47^c^	2.74±2.08^c^
F/p				**8.484/0.000** *	**382.675/0.000** *	**402.741/0.000** *
Nutritional status after discharge	Breastmilk only	15	13.3	93.73±10.94^a^	42.00±21.21^b^	10.87±10.43^b^
	Formula milk only	67	59.3	83.07±10.37^b^	61.43±16.29^a^	18.90±7.79^a^
	Breastmilk and formula milk	31	27.4	85.84±8.83^b^	67.97±12.96^a^	21.00±6.47^a^
F/p				**6.968/0.001** *	**13.191/0.000** *	**8.729/0.000** *
Length of stay in the NICU	<3 months	43	38.1	88.56±11.84^a^	49.12±20.94^b^	12.86±9.58^b^
	>4 months	70	61.9	83.21±9.22^b^	67.73±10.85^a^	21.81±5.21^a^
t/p				**2.527/0.014** *	**-5.400/0.000** *	**-5.637/0.000** *

a,b,c significant mean differences between the groups (a: the highest mean), F: one-way ANOVA test, t: independent sample t-test,

* p<0.05. SD: standard deviation; MAS: Maternal Attachment Scale; MICBS: Mother-Infant Contact Barriers Scale; EPDS: Edinburgh Postnatal Depression Scale; NICU: neonatal intensive care unit. Statistically significant values are denoted in bold.

It was found that mothers who had a planned pregnancy, who gave birth between the 33rd and 37th weeks of gestation, and whose postpartum period was less than 3 months had higher MAS scores, while MICBS and EPDS score averages increased as the gestational week decreased and whose postpartum period was 4 months or more (p<0.05). The mean MAS score of mothers whose infants were exclusively breastfed was higher compared to mothers whose infants were fed with ready-made formula or breast milk+formula, and the mean MICBS and EPDS scores of mothers whose infants were fed with ready-made formula or breast milk+formula were higher than those of mothers whose infants were exclusively breastfed (Table 1).

The mean EPDS score of 78.8% (n=89) of the mothers was 14 and above. A statistically significant negative relationship (p<0.05) was revealed between the mothers’ MAS and MICBS scores and the postpartum physical contact barriers, mother-infant relationship and harmony difficulties, and positive emotions regarding postpartum first contact sub-scales and the EPDS. Accordingly, it can be said that maternal attachment decreases as maternal and infant contact barriers and PND increase (Table 2). There was a statistically significant positive relationship between the mean MICBS and EPDS scores of the participating mothers in the study (p<0.05). Therefore, it can be stated that as mother and infant contact barriers increase, PND also increases (Table 2).

**Table 2:** Relationship between scales and sub-dimensions.

Variables		MAS	MICBS	Postpartum physical contact barriers	Mother-infant relationship and harmony difficulties	Negative experiences regarding birth	Positive emotions regarding postpartum first contact	EPDS
MAS	r	–	-0.316	-0.342	-0.363	0.003	-0.335	-0.286
	p		**0.001** **	**0.000** **	**0.000** **	0.977	**0.000** **	**0.002** **
MICBS	r		–	0.888	0.923	0.674	0.793	0.880
	p			**0.000** **	**0.000** **	**0.000** **	**0.000** **	**0.000** **
Postpartum physical contact barriers	r			–	0.761	0.532	0.790	0.770
	p				**0.000** **	**0.000** **	**0.000** **	**0.000** **
Mother-infant relationship and harmony difficulties	r				–	0.398	0.693	0.871
	p					**0.000** **	**0.000** **	**0.000** **
Negative experiences regarding birth	r					–	0.374	0.489
	p						**0.000** **	**0.000** **
Positive emotions regarding postpartum first contact	r						–	0.701
	p							**0.000** **

r: Pearson's correlation analysis,

** p<0.001. Statistically significant values are denoted in bold. EPDS: Edinburgh Postnatal Depression Scale, MICBS: Mother-Infant Contact Barriers Scale, MAS: Maternal Attachment Scale.

The MICBS had a statistically significant positive effect on the EPDS (B=0.95, p<0.001), and the EPDS had a statistically significant negative effect on the MAS (B=-0.29, p<0.001). Furthermore, it was found that the EPDS played a mediating role (B=-0.27, p<0.001) in the impact of the MICBS on the MAS. The Goodness of Fit Index (0.925), Tucker-Lewis Index (0.912), Normed Fit Index (0.937), Incremental Fit Index (0.954), Comparative Fit Index (0.953), and Standardized Root Mean Square Residual (0.063) values of the path model were acceptable^15,16^.

## DISCUSSION

Concerning mother-infant contact barriers, preterm birth becomes important since it often causes the mother and the infant to be separated from each other. Preterm birth is among the most common obstetric complications in the peripartum period and is one of the leading causes of neonatal deaths^6,7^. Preterm birth is related to numerous negative outcomes for both the mother and infant, such as increased psychological stress with care difficulties for parents and cognitive impairments in the child^7,8^. This study, which was carried out to evaluate the correlation between PND, mother-infant contact barriers, and maternal attachment in mothers of preterm infants discharged from the hospital, showed that maternal attachment decreased and PND increased as mother-infant contact barriers increased and determined that PND played a mediating role in the effect of preventing the mother and infant contact on maternal attachment.

The mother's psychological health represents a significant factor that may pave the way for mother-infant contact barriers and, thus, insecure attachment. In the literature review, it has been stated that childbirth and the subsequent puerperium may predispose to psychological problems that may be experienced in the postpartum period^8,17^. Studies indicate that various psychological health problems, e.g., psychosis, depression, anxiety disorders, or the comorbidity of these psychological problems, can be observed in mothers after childbirth^7,9,17,18^. It is also known that mothers who have given preterm birth are at higher risk than mothers who have given birth at term^5,19^. In addition, preterm birth can cause feelings in mothers such as failure to maintain the pregnancy, sadness at the loss of the imagined baby, helplessness, anxiety and fear for the baby's future ^17,20^. Psychological problems experienced after childbirth can impact the mother's relationship with her infant and her contact with her infant, causing long-term negative impacts in terms of maternal and infant health^1,21^.

Upon examining the differences between the sociodemographic variables and the scales that make up another dimension of the study, it was found that those whose infants spent more than 4 months in the neonatal intensive care unit after birth, mothers of infants with a low gestation week and weight, and mothers feeding their infants with ready-made formula were more disadvantaged compared to other groups and usually had low maternal attachment levels and high contact barriers and depression levels. In a systematic review by Darvishvand et al. examining 27 articles, the factors affecting maternal attachment were divided into two regarding the mother and the infant^22^. In this study, whereas maternal factors were listed as age, education level, job, income, place of residence, psychosocial support, marital satisfaction, psychological disorders, the number of children, the number of pregnancies, unplanned pregnancies, high-risk pregnancies, prenatal attachment level, mode of delivery, staying in the same room with the infant, skin-to-skin contact, effective communication, mother's perception of the infant, involvement in newborn care, and early initiation of breastfeeding, the infant-related factors were determined as health problems, prematurity, infant's sex, and birth weight. Another important result of our study is exclusive breastfeeding rates in preterm infants discharged from the hospital. It was observed that 15 of the mothers included in the study exclusively breastfed their infants. In the literature, the relationship between breastfeeding and PND has been conceptualized as one-way, and it has been revealed that PND adversely impacts the rates of initiating and discontinuing breastfeeding^23,24^. Furthermore, there are some pieces of evidence indicating that breastfeeding may protect against PND or help improve symptoms faster^24-26^.

Studies on mother-infant contact barriers are limited^10^. However, a meta-analysis study reveals that stressful life events are predictors of PND^7^. Considering that mother-infant contact barriers also involve stressful experiences, the results of our research and the high level of depression in mothers confirm the cause and effect relationship between the two. Hence, there is a need for long-term studies involving different sample groups on this subject.

## CONCLUSION

It has been determined that maternal attachment may be adversely impacted by the mother's psychological/social condition in the postpartum period and when the mother's contact with her infant is prevented, e.g., preterm birth. Healthcare professionals must be aware of the factors that may adversely impact mother-infant contact. The follow-up of risky pregnant women, basic education, and counseling services to improve motherhood skills should be planned and implemented starting from the antenatal period.

## ETHICAL CONSIDERATIONS

The study was approved by the Clinical Studies Ethics Committee of Kahramanmaraş University in Turkey (approval number: 03, date: 22.03.2022), and institutional permission was obtained. The study adheres to the principles of the Helsinki Declaration. All subjects provided informed consent prior to data collection.

## LIMITATIONS

The present research has some limitations. First, the data acquired are limited to mothers of 1-4-month-old preterm infants and cannot be generalized to all periods. Additionally, the data were evaluated in line with the personal answers given to the questions included in the measurement tool.
